# New onset or recurrence of uveitis following COVID-19 infection

**DOI:** 10.1186/s12886-024-03289-w

**Published:** 2024-01-17

**Authors:** Hui Feng, Meng Zhao, Jing Mo, Xusheng Cao, Weixin Chen, Hong Wang

**Affiliations:** Beijing Ophthalmology and Visual Sciences Key Laboratory, Beijing Tongren Eye Center, Beijing Institute of Ophthalmology, Beijing Tongren Hospital, Capital Medical University, 1 Dongjiaominxiang Street, Dongcheng District, Beijing, China

**Keywords:** COVID-19, New onset uveitis, Uveitis relapse

## Abstract

**Background:**

While the 2019 novel coronavirus disease (COVID-19) pandemic has resulted in millions of cases worldwide, there is increasing recognition of a wide range of ocular manifestations associated with the virus, including uveitis. Uveitis is an inflammatory condition of the uveal tract of the eye that can lead to permanent vision loss if not treated promptly. Here we report a retrospective observational study of patients who presented with new onset or recurrent uveitis following COVID-19 infection.

**Methods:**

This is a retrospective observational study conducted at the Beijing Tongren Hospital. We identified patients who presented with symptoms of non-infectious active uveitis with positive real-time reverse transcription polymerase chain reaction (RT-PCR) of COVID-19 within 4 weeks. All patients received ophthalmic examinations, including anterior and posterior segment imaging, to assess the extent of ocular involvement.

**Results:**

The 18 patients with a total of 33 eyes included in this study presented with symptoms of active uveitis within 4 weeks of their positive COVID-19 RT-PCR test. Among them, 9 patients presented with the development of uveitis following COVID-19 infection, and 9 patients had relapsed uveitis after COVID-19 infection. Treatment with corticosteroids resulted in improvement of symptoms and resolution of inflammation in all cases. In this study, all patients did not experience any adverse drug reactions during treatment.

**Conclusion:**

Our observational study highlights the potential for new onset or recurrence of uveitis following COVID-19 infection.

**Trial registration:**

https://www.chictr.org.cn/; identifier: ChiCTR2100044365, date: 03/17/2023.

## Background

In December 2019, the outbreak of the 2019 novel coronavirus disease (COVID-19) caused by severe acute respiratory syndrome coronavirus 2 (SARS-CoV-2) occurred in China [1]. During the COVID-19 pandemic, which swept across the globe, attention was focused on patients with various clinical features of inflammatory syndromes. Numerous studies have shown that COVID-19 infection can cause a systemic inflammatory response characterized by life-threatening excessive inflammation sustained by cytokine storms, ultimately leading to multi-organ failure [2]. The extent of these inflammatory reactions is not yet fully understood, but ophthalmologists have observed that the coronavirus can also produce a wide range of ocular manifestations, from anterior segment involvement such as conjunctivitis and anterior uveitis [3–5], to posterior segment diseases such as retinitis and optic neuritis involving the retina and choroid [6, 7].

Uveitis is a collection of diseases characterized by inflammation within the eye, and its etiology is multifactorial, including autoimmune (60.1%), systemic (30–50%), infectious (30–50%), and idiopathic (20–40%) causes [8]. The relationship between viral infections and uveitis is extremely complex and not fully understood at present. Immune system alterations triggered by coronavirus infections may impact various forms of inflammation, including uveitis [9]. It has been observed that the incidence of uveitis increased significantly during the COVID-19 pandemic [10]. Additionally, SARS-CoV-2 can induce retinal lesions or retinal vascular changes. Whether SARS-CoV-2 can cause distinct retinitis, similar to retinitis caused by herpes viruses, remains to be confirmed in larger series studies [11].

Following the resurgence of COVID-19 at the end of 2022, we observed a significant increase in the number of patients presented with the recurrence of uveitis following COVID-19 infection., and some patients presented with new-onset uveitis as well. We conducted a retrospective observational study on this group of patients, aiming to investigate in depth the relationship and pathogenesis between COVID-19 infection and uveitis, and understand the characteristics of the occurrence of uveitis after COVID-19 infection through their clinical presentation and features.

## Methods

This is a retrospective observational study conducted at the Beijing Tongren Hospital. The study included patients diagnosed with non-infectious acute intraocular inflammation from December 1, 2022, to February 28, 2023, who had a history of COVID-19 infection within one month. The study was approved by the institutional review board and ethics committee of Beijing Tongren Hospital and was conducted in accordance with the principles of the Helsinki Declaration.

The inclusion criteria of this study were: (1) patients diagnosed with non-infectious active uveitis according to the Standardization of Uveitis Nomenclature (SUN) Working Group classification system [12]. Patients with active inflammation had at least one of the following: ≥1 + anterior chamber cell/flare, ≥ 1 + vitreous haze, chorioretinal inflammation, retinal vasculitis, or optic neuritis. (2) positive real-time reverse transcription polymerase chain reaction (RT-PCR) for SARS-CoV-2 obtained from nasopharyngeal swabs, and (3) a close temporal relationship between the onset of the disease and the diagnosis of SARS-CoV-2 infection, i.e., within one month. Patients whose ocular inflammation was reactivated due to inadequate treatment or discontinuation of medication were excluded.

The researchers reviewed the clinical records of the included patients to obtain demographic data and ocular history, with particular attention to the duration between COVID-19 diagnosis and the onset of visual symptoms. All patients underwent comprehensive ocular examinations, including best-corrected visual acuity, anterior segment examination with a slit-lamp (BQ900, Haag-Streit, Bern, Switzerland), intraocular pressure (IOP) (NT510, Non-Contact tonometer, Nidek, Gamagori, Japan), and indirect ophthalmoscopy. In addition, relevant imaging studies were performed, including fundus photography (CR-DGi Non-mydriatic retinal camera, Canon, Tokyo, Japan), B-scan ultrasound (Echoscan, US-400; Nidek Co Ltd, Gamagori, Japan), fluorescein angiography (FFA), Indocyanine green angiography (ICGA) (Spectralis HRA; Heidelberg Engineering, Inc, Heidelberg, Germany) and spectral domain optical coherence tomography (SD-OCT) (RTVue, Optovue, Inc, CA). A laboratory workup was conducted for all patients to exclude infection and evaluate systemic conditions, which included routine blood and urine tests, liver and kidney function analysis, infectious disease investigations (including hepatitis serology, and screening for syphilis and HIV antibodies). All patients underwent screening for tuberculosis; Purified Protein Derivative (PPD) and QuantiFERON-TB Gold test (QFT).

All patients had complete medical records, including gender, age, medical history, classification of uveitis according to the SUN Working Group classification, treatment plans, treatment outcomes, and their conditions at the time of their last visit. All statistical data in this study were entered into Excel. The continuous variables were described as means and standard errors of the mean (SEM). The categorical variables were described as frequencies and constituent ratios.

## Result

This retrospective observational study, based on a retrospective hospital-based observational study, included 18 patients with a total of 33 eyes. Among them, 9 patients presented with the development of uveitis following COVID-19 infection, and 9 patients had relapsed uveitis after COVID-19 infection.

### New onset uveitis

Among the newly diagnosed uveitis patients, there were 4 male and 5 female patients, with an average age of 37.6 ± 15.8 years. Except for recent COVID-19 infection, these patients had no history of other systemic or ocular diseases. Of the 9 newly diagnosed uveitis patients in this study, 7 had bilateral involvement. According to the SUN Working Group classification [12], 3 patients had Vogt-Koyanagi-Harada (VKH) syndrome, 1 had sympathetic ophthalmia, 4 had anterior uveitis, and 1 had multiple evanescent white dot syndrome (MEWDS). Table 1 provides detailed demographic data of all patients with clinical features.

**Table 1:** Demographic and clinical data of the new onset uveitis patients

Patient number (age/sex/eye)	Time from COVID − 19 RT-PCR to uveitis (day)	Etiology	Clinical examination on admission		Treatment	Duration of follow up (day)	Outcome at last follow-up	
			BCVA	IOP			BCVA	IOP
Patient 1 (47/F/OU)	2	Vogt-Koyanagi-Harada disease	CF; CF	19.1, 18.5	Topical Steroids Oral Steroids Ciclosporin	65	0.3, 0.6	20.1, 19.2
Patient 2 (29/F/OU)	1	Vogt-Koyanagi-Harada disease	HM; CF	9.9, 9.3	Topical Steroids Oral Steroids Ciclosporin	58	0.2, 0.3	11.3, 11.4
Patient 3 (27/M/OU)	14	Vogt-Koyanagi-Harada disease	CF; CF	10.1, 12.5	Topical Steroids Oral Steroids Ciclosporin	60	0.1, 0.5	12.4, 12.2
Patient 4 (25/M/OU)	2	Sympathetic Ophthalmia	HM, 0.2	14.1, 12.4	Oral Steroids	53	NLP, 0.3	10.3, 18.8
Patient 5 (38/F/OU)	7	Anterior Uveitis	0.2, 0.4	13.6, 14.6	Topical Steroids Peribulbar methylprednisolone injection	34	0.3, 1.0	11.3, 14.5
Patient 6 (36/F/OD)	20	Anterior Uveitis	0.8, 1.0	13.4, 15.6	Topical Steroids Peribulbar methylprednisolone injection	55	1.0, 1.0	12.4, 12.6
Patient 7 (58/M/OU)	13	Anterior Uveitis	0.8, 0.8	18.9, 18.2	Topical Steroids Peribulbar methylprednisolone injection	92	0.8, 0.8	16.4, 17.1
Patient 8 (46/M/OU)	7	Anterior Uveitis (Posner-Schlossman Syndrome)	0.6, 0.2	32.4, 30.8	Topical Steroids IOP-lowering medication Trabeculectomy surgery	90	0.8, 0.6	12.4, 16.4
Patient 9 (31/F/OS)	7	Choroiditis	1.0, 0.4	13.2, 12.3	Topical Steroids, Oral Steroids	62	1.0, 1.0	14.4, 14.1

*Abbreviations*: BCVA, best corrected visual acuity; IOP, intraocular pressure; HM, hand movement; CF, counting Finger

**Fig. 1:**
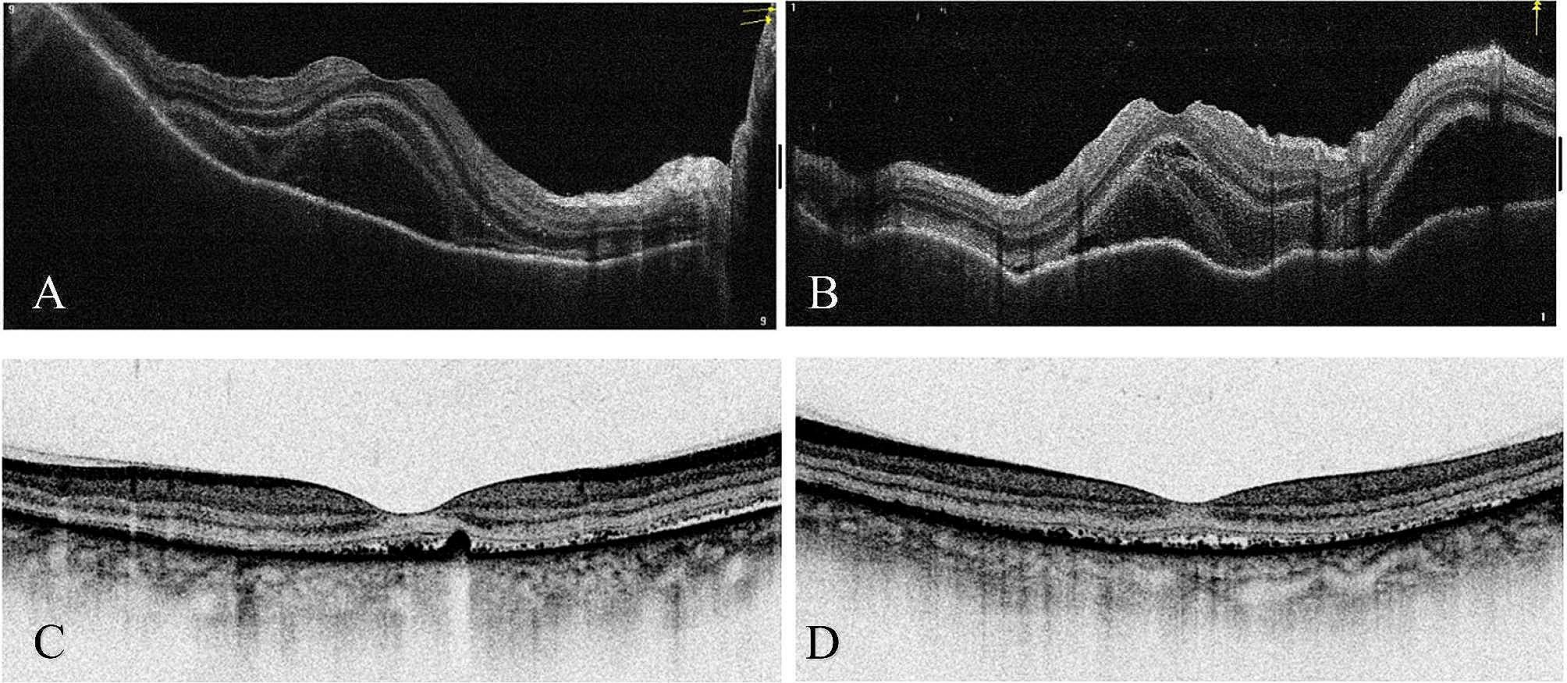
At the onset of VKH, SD-OCT examination revealed exudative retinal detachment at the macula of both eyes (**A**: right eye; **B**: left eye). After treatment with oral corticosteroids and immunosuppressive agents, a follow-up SD-OCT examination one month later showed disappearance of the exudative retinal detachment in both eyes (**C**: right eye; **D**: left eye). VKH: Vogt-Koyanagi-Harada syndrome; SD-OCT: spectral domain optical coherence tomography

Three patients who developed VKH for the first time all presented with bilateral eye redness, decreased vision, and headache within 2 weeks after COVID-19 infection. Upon examination under a slit lamp, both eyes had conjunctival hyperemia, ciliary injection, cornea with fine and moderate keratic precipitates, and cellularity and flare in the anterior chamber. In addition, SD-OCT examination of the posterior segment of all three patients revealed extensive exudative retinal detachment involving the macula in both eyes. After treatment with oral corticosteroids and immunosuppressive agents, the vision of all VKH patients improved compared to the onset of the disease, and OCT examination showed disappearance of exudative retinal detachment in both eyes. Figure 1 shows patient 2, in whom SD-OCT examination revealed exudative retinal detachment at the macula in both eyes at onset of VKH. After treatment with oral corticosteroids (1 mg/kg/day), immunosuppressive agents (cyclosporine 3 mg/kg/day), and topical prednisolone 1%, follow-up SD-OCT examination after 1 month showed the disappearance of exudative retinal detachment in both eyes.

We also found a patient who developed sympathetic ophthalmia 2 days after being infected with COVID-19. Patient 4 had undergone a scleral laceration repair surgery in the right eye due to being hit by an iron rod 3 months prior, and later underwent a pars plana vitrectomy (PPV) and silicon oil filling in the right eye one month later. After experiencing symptoms of upper respiratory tract infection and testing positive for COVID-19 on PCR testing, the patient noticed a decrease in vision in both eyes 2 days later. Fundus examination showed choroidal and scleral exposure in the right eye, a mass-like retinal tissue in front of the optic disc in the right eye, and serous retinal detachment at the macula in the left eye. SD-OCT examination revealed retinal atrophy and thinning in the right eye, retinal detachment at the macula, and serous retinal detachment at the macula in the left eye with accompanying detachment of the retinal pigment epithelium. After receiving oral corticosteroid treatment (1 mg/kg/day), the subretinal fluid in the left eye decreased, but the retinal detachment in the right eye did not resolve. At the patient’s last follow-up visit, the patient had lost vision in the right eye, but had improved vision in the left eye compared to onset.

Within 1–3 weeks after being diagnosed with COVID-19 infection, four patients developed anterior uveitis and sought medical attention due to eye redness. These patients denied having a history of chronic autoimmune and inflammatory diseases. Slit-lamp examination revealed conjunctival congestion, anterior chamber flare, and cornea with fine and moderate keratic precipitates in all patients. After treatment with topical prednisolone acetate 1% (starting at 8 times a day and reducing every two days) and posterior subtenon triamcinolone injections, all patients responded well to the medication and had improved vision. One patient developed elevated intraocular pressure and secondary glaucoma, which could not be controlled with oral and topical IOP-lowering medication. Therefore, trabeculectomy was performed in both eyes, and the patient’s intraocular pressure normalized after surgery. Following the surgical intervention, He was started on oral valganciclovir 900 mg two times a day for 6 weeks and then one time a day for 6 weeks, topical prednisolone acetate (1%) six times a day and tapered every week and topical antibiotics for the first 2 weeks. At 3-month follow-up, a well-formed bleb was seen and the IOP was controlled within the normal range. Furthermore, the patient’s BCVA was 0.8 in the right eye and 0.6 in the left eye and signs of the anterior uveitis were absent.

**Fig. 2:**
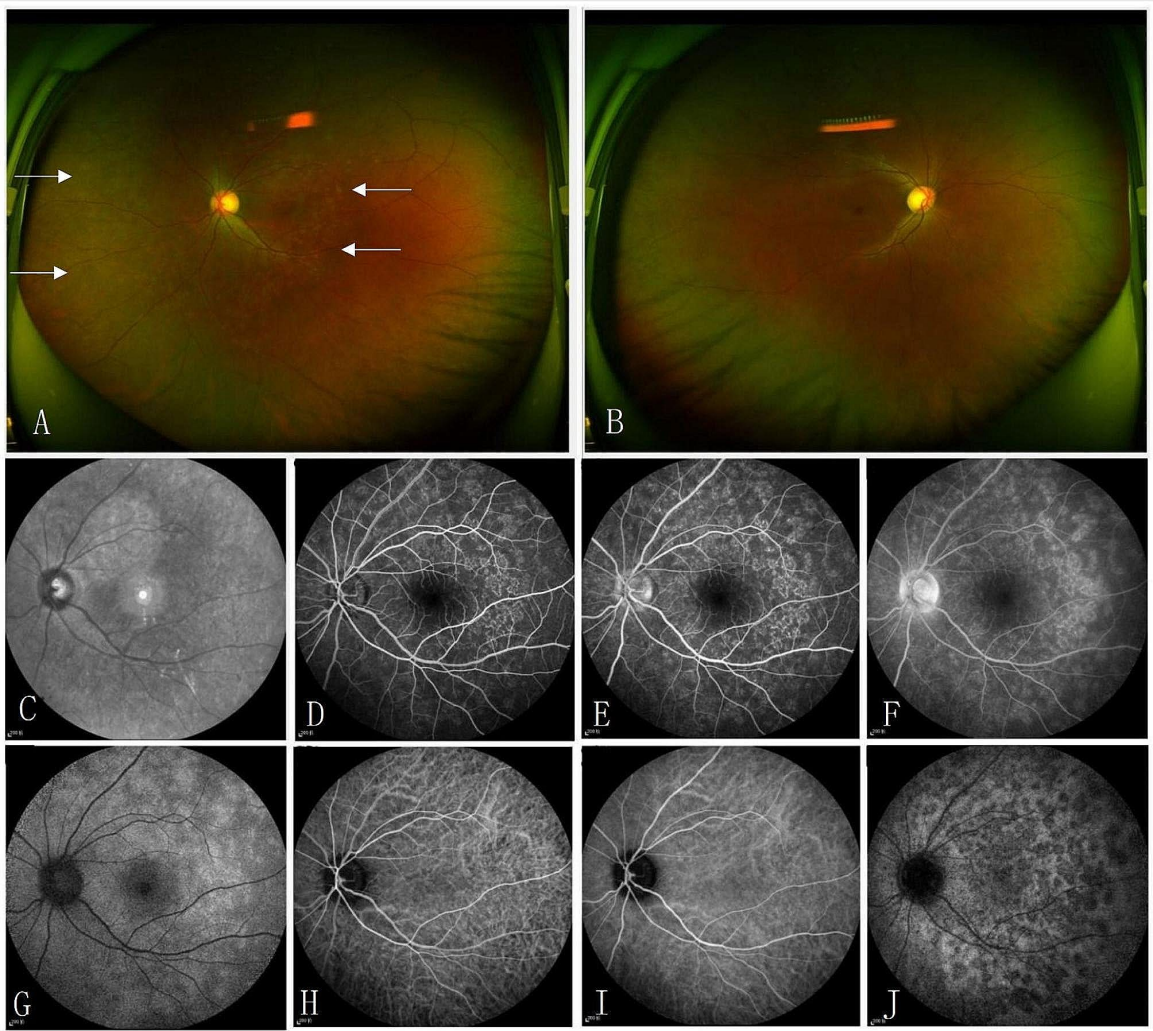
Patient 9’s multimodal imaging showed characteristics of MEWDS. Wide-angle fundus photography of the left eye revealed multiple yellow lesions distributed on the retina (arrows) (**A**, **B**). Flaky hyperfluorescent on the temporal side of the optic disc was observed in the infrared fundus image. (**C**)Early-phase FFA showed scattered punctate hyperfluorescence (**D**), while mid-to-late phase showed staining around the optic disc with blurred margins arranged in a wreath-like pattern, consistent with the location of spontaneous fluorescence (**E**, **F**). FAF exhibited various-sized patchy areas of hyperfluorescence near the optic disc and posterior pole as well as uneven diffuse hyperfluorescence (**G**). ICGA showed normal choroidal background fluorescence in early-phase with no abnormality observed in the large and medium vessels. In the mid-to-late phase, multiple scattered weakly fluorescent lesions were observed in the choroid around the optic disc, macular area, and mid-periphery, varying in size, and some lesions fused into small patches. (**H**–**J**) MEWDS: multiple evanescent white dot syndrome; FFA: fluorescein angiography; ICGA: indocyanine green angiography

Patient 9 was a 31-year-old female who presented with gradual loss of vision in her left eye. It is worth noting that she was diagnosed with COVID-19 one week prior. The patient’s primary COVID-19 symptoms included malaise, cough, and mild fever, and she had no significant medical history. HIV, syphilis, tuberculosis, and sarcoidosis were ruled out. Complete blood count, C-reactive protein, and erythrocyte sedimentation rate were within normal limits. Chest X-ray was normal. Fundus autofluorescence (FAF) and SD-OCT examination showed no significant differences in the right eye. In the left eye, FAF revealed multiple, small, and widely distributed hyper-autofluorescent lesions in the outer retina (Fig. 2), and SD-OCT showed disruption of the ellipsoid zone (EZ) and hyper-reflective material extending through the EZ into the outer nuclear layer. FFA showed no significant lesions in the right eye, while diffuse high fluorescence of retinal lesions and optic disc fluorescence enhancement were observed in the left eye (Fig. 2). ICGA showed no significant lesions in the right eye, while multiple scattered weak fluorescence lesions were observed in the left eye (Fig. 2). These findings are consistent with the diagnosis of MEWDS, confirmed by further examinations and investigations. This patient received prednisolone acetate tablets at a daily dosage of 40 mg, which was subsequently reduced by 5 mg every four days. In addition to systemic steroid treatment, the patient received tobramycin dexamethasone eye drops four times a day for a duration of two weeks. After local and systemic steroid treatment for one month, the patient’s vision improved to 1.0, and the white dot lesions in the fundus disappeared.

### Recurrent uveitis

The study included 4 male and 5 female patients with a mean age of 39.9 ± 16.8 years. Among the 9 patients presented with uveitis recurrence in this study, 8 patients had bilateral involvement. According to the SUN Working Group classification criteria for uveitis [12], 3 cases were diagnosed with VKH syndrome, 3 cases with anterior uveitis, 2 cases with intermediate uveitis, and 1 case with panuveitis. Table 2 details all demographic data with clinical characteristics.

**Table 2:** Demographic and clinical data of the with the patients recurrent uveitis

Patient number (age/sex/eye)	Time from COVID-19 diagnosis to uveitis (day)	Etiology	Clinical examination on admission		Treatment	Duration of follow up (day)	Outcome at last follow-up	
			BCVA	IOP			BCVA	IOP
**Adult patient**								
Patient 10 (53/F/OU)	20	Vogt-Koyanagi-Harada disease	0.3, 0.25	13.6, 11.5	Topical Steroids Peribulbar methylprednisolone injection Oral Steroids, Ciclosporin	58	0.4, 0.5	14.5, 13.4
Patient 11 (59/M/OU)	21	Vogt-Koyanagi-Harada disease	0.2, 0.2	14.6, 17.1	Topical Steroids Peribulbar methylprednisolone injection Oral Steroids	66	0.8, 0.8	17.6, 17.5
Patient 12 (54/M/OU)	7	Vogt-Koyanagi-Harada disease	0.1, 0.1	17.9, 21.8	Topical Steroids Peribulbar methylprednisolone injection Oral Steroids, Ciclosporin	54	0.1, 0.2	13.1, 12.8
Patient 13 (33/M/OU)	5	Anterior Uveitis	0.6, 0.7	12.9, 13.7	Topical Steroids	34	0.9, 1.0	13.1, 14.7
Patient 14 (63/M/OS)	17	Anterior Uveitis (Posner-Schlossman Syndrome)	0.7, 0.1	14.3, 38.3	Topical Steroids IOP-lowering medication,	65	0.8, 0.6	15.4, 16.4
Patient 15 (35/F/OU)	12	Intermediate Uveitis	0.9, 0.9	17.7, 18.6	Topical Steroids Peribulbar methylprednisolone injection Oral Ciclosporin Subcutaneous Injection of Adalimumab	64	1.0, 1.0	12.4, 16.4
Patient 16 (24/F/OU)	14	Panuveitis	0.1, 0.3	11.5, 16.5	Topical Steroids Subconjunctival injection of dexamethasone Oral Steroids Mycophenolate Mofetil Subcutaneous Injection of Adalimumab	55	0.2, 0.6	14.4, 13.5
**Pediatric patient**								
Patient 17 (11/M/OU)	7	Anterior Uveitis (Juvenile Idiopathic Arthritis-associated Uveitis)	1.2, LP	14.1, 38.3	Topical Steroids Oral Steroids Mycophenolate Mofetil Subcutaneous Injection of Adalimumab Peripheral Iridectomy	92	1.2, LP	13.3, 20.8
Patient 18 (12/F/OU)	21	Intermediate Uveitis	0.7, 0.7	13.7, 16.5	Topical Steroids Peribulbar methylprednisolone injection Oral Mycophenolate Mofetil Subcutaneous Injection of Adalimumab	92	1.0, 1.2	14.4, 13.1

*Abbreviations*: BCVA, best corrected visual acuity; IOP, intraocular pressure; HM, hand movement; CF, counting Finger; LP, light perception

Three patients with recurrent VKH syndrome had previously maintained stable inflammation for over six months with prednisolone acetate tablets (less than 10 mg/day) and cyclosporine (50 mg/day), but after being diagnosed with COVID-19 infection 1–3 weeks earlier, they presented with bilateral vision loss and were reexamined. These three patients primarily presented with symptoms of COVID-19, including fever, sore throat, and persistent dry cough. Slit-lamp examination revealed anterior uveitis relapse, keratic precipitates and flare in the anterior chamber of three patients. We administered prednisolone acetate eye drops 1% four times a day for two weeks, along with peribulbar injections of methylprednisolone 20 mg a total of two times, once every other day to patients’ treatment regimen based on oral steroids and immunosuppressants. Upon follow-up, anterior uveitis was well-controlled in all 3 VKH patients, and no ocular complications were observed.

**Fig. 3:**
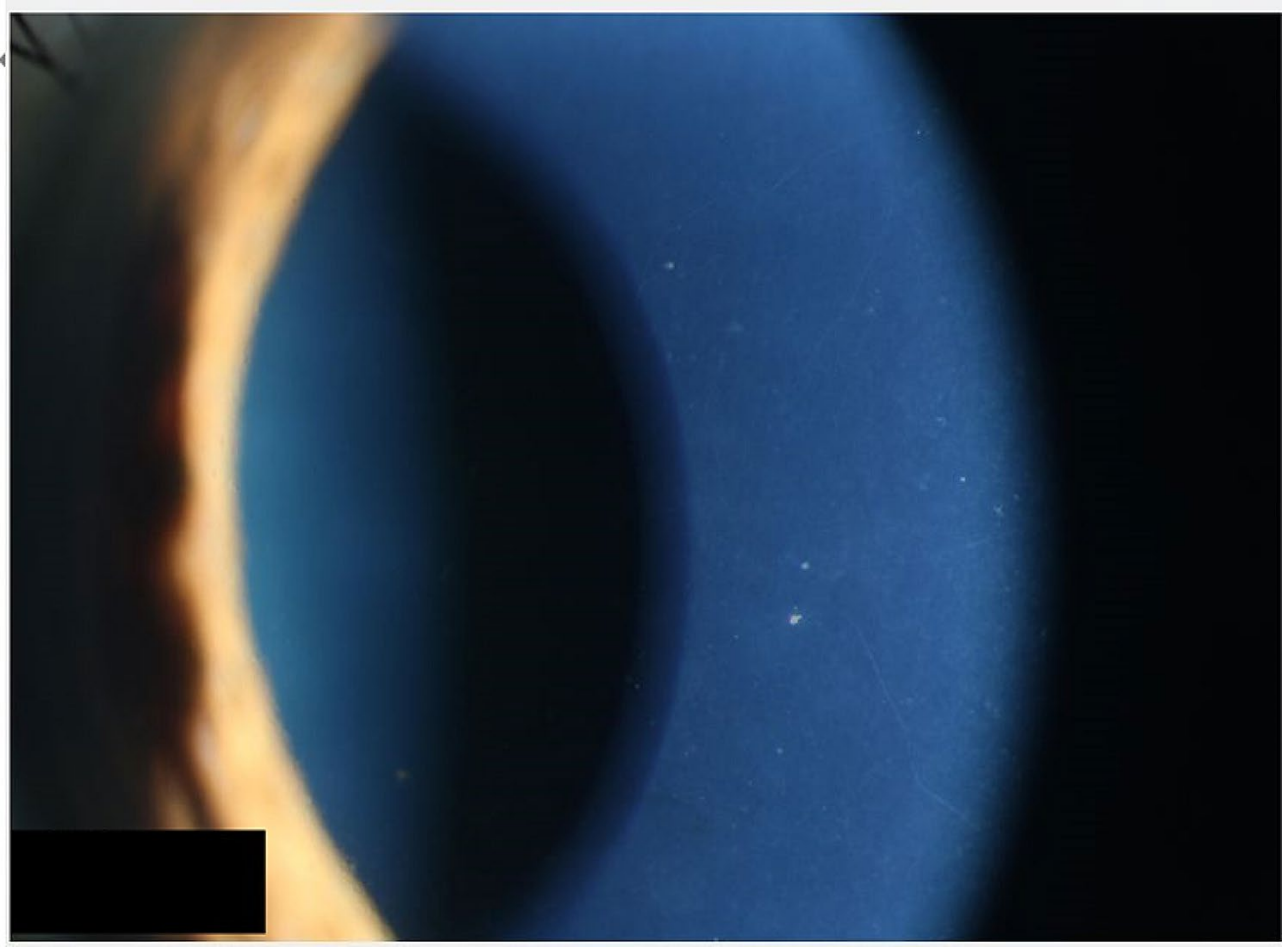
During the eye examination of patient 14, white keratic precipitates were observed behind the cornea, and inflammation recurred

Among the 3 patients with anterior uveitis, 2 patients had elevated intraocular pressure. Patient 17 had anterior uveitis caused by juvenile idiopathic arthritis (JIA)-associated uveitis since the age of 4 years old. The patient’s ocular inflammation was well-controlled with oral methylprednisolone tablets at a dosage of 4 mg per day, immunosuppressants (mycophenolate mofetil tablets 50 mg per day), and subcutaneous injections of adalimumab (40 mg per injection, every two weeks). The visual acuity of both eyes was maintained at 1.0. One month ago, patient 17 was diagnosed with COVID-19 and complained of decreased vision and eye pain in the left eye. The patient’s COVID-19 symptoms primarily manifested as fever and dry cough. Ophthalmic examination revealed vision decreased to light perception and IOP elevated in the left eye. Slit-lamp examination revealed pigmentary keratic precipitates in the anterior chamber of the left eye, iris posterior synechiae, and mutton-fat keratic precipitates. Fundus examination showed pallor of the left optic disc with a C/D ratio of 0.9. We immediately performed peripheral iridectomy on the left eye. Postoperative follow-up showed well-controlled intraocular pressure, but the visual acuity did not improve. Patient 14 had anterior uveitis caused by Posner-Schlossman syndrome (PSS) associated with glaucomatocyclitic crisis, and the inflammation and intraocular pressure were well-controlled for 8 months with topical prednisolone acetate three times daily and timolol maleate eye drops twice daily. Three weeks ago, patient 14 was diagnosed with COVID-19 and complained of decreased vision and eye pain in the left eye. Ophthalmic examination revealed elevated IOP and KP in the anterior chamber of the left eye (Fig. 3). We added brinzolamide eye drops twice daily, bromfenac eye drops three times daily, latanoprost eye drops once daily, and ganciclovir eye drops four times daily to the treatment regimen of patient 14. Upon reexamination one month later, the intraocular pressure had returned to normal.

We observed recurrences of inflammation in two intermediate uveitis patients and one panuveitis patient. The two intermediate uveitis patients had previously achieved disease stability through oral immunosuppressants (Cyclosporin soft capsules 25 mg per day) and subcutaneous injections of adalimumab (40 mg per injection, every two weeks), while the panuveitis patient had achieved stability through oral prednisolone acetate tablets at a dosage of 5 mg per day, immunosuppressants (mycophenolate mofetil tablets 25 mg per day), and subcutaneous injections of adalimumab (40 mg per injection, every two weeks). All three patients had stable disease control for more than 6 months. Within 2–3 weeks after COVID-19 infection, all three patients experienced decreased visual acuity in both eyes, and slit-lamp examination revealed cells in anterior vitreous. After treatment with prednisolone acetate eye drops 1% four times a day for two weeks, and peribulbar injections of methylprednisolone 20 mg a total of two times, once every other day, the anterior vitreous cells disappeared and the inflammation was controlled.

## Discussion

SARS-CoV-2 has been detected in ocular secretions, although the exact pathophysiology of ocular transmission of the virus remains unclear. The potential for this virus to cause local ocular disease is worth considering [13]. This retrospective observational study found that COVID-19 infection can cause the onset and recurrence of autoimmune uveitis. Inflammation can be stabilized and vision can be partially restored through corticosteroids therapy.

The mechanisms underlying uveitis within the context of viral infections, notably SARS-CoV-2, pivot on complex interactions involving the virus, the immune system, and the ensuing inflammatory responses. Systemically, SARS-CoV-2 infection is divided into two phases, with the initial phase reflecting direct viral responses and the later phase marked by an autoimmune-inflammatory reaction. Initially, In SARS-CoV-2 infection, two types of immune responses are involved. The innate immune system provides the initial defense against the virus, and then adaptive immune response, involving cytotoxic T cells and B cells producing neutralizing antibodies [14].

In stark contrast, the latter phase mirrors the features of an autoimmune ailment, driven by a cytokine storm and a vigorous inflammatory response. For individuals in the post-COVID-19 phase, this phase can potentially heighten the systemic inflammatory burden, consequently serving as a potential trigger for ocular inflammation. Normally, an adequate interferon (IFN) response induces an antiviral state in infected cells, limiting viral replication and protecting the host [15, 16]. However, some SARS-CoV-2 proteins can suppress antiviral type I IFN production and signaling. This initial delay in the IFN response can result in unrestrained viral replication and dissemination, followed by an eventual increase in IFN, exacerbating inflammation [17, 18]. This phenomenon is attributed, in part, to “molecular mimicry” and “bystander activation.” An exuberant antiviral immune response fosters a localized pro-inflammatory milieu, instigating the liberation of self-antigens from damaged tissues. Subsequently, these self-antigens are recognized and presented by antigen-presenting cells, thereby activating auto-reactive T cells, ultimately triggering autoimmunity through the process termed “bystander activation” [19].

Clinical and ocular manifestations of COVID-19 infection and its complications may vary and fluctuate, while there are limited widespread research. A recent Meta-analysis showed 11.64% of COVID-19 patients had ocular surface manifestations [20]. Common ocular presentations of COVID-19 include eye pain, redness, and follicular conjunctivitis. However, despite the detection of SARS-COV2 in the blood during acute COVID-19 infection and the presence of ACE2 receptors in ocular tissues, uveitis following COVID-19 is not commonly encountered [21]. Nonetheless, uveitis, if not promptly detected and treated, can significantly impact patient’s life. Uveitis can lead to complications such as cataracts, posterior adhesions, glaucoma, optic nerve swelling, resulting in decreased or even lost vision. In our study, among the 7 cases of new or recurrent anterior uveitis, 3 patients developed high intraocular pressure. 2 patients received timely drug or surgical treatment and did not suffer serious vision damage. However, in one case, after COVID-19 infection, anterior uveitis relapsed, with adhesion between the iris and lens, causing raised IOP. The patient did not receive timely treatment to lower the intraocular pressure, resulting in permanent vision damage. Alonso reported a case of a 62-year-old man who developed anterior uveitis after COVID-19 infection, leading to increased intraocular pressure [3]. Clinical treatment was ineffective, and surgical intervention was performed. The patient’s vision improved from a 1-meter index to 20/50. Vienne-Jumeau summarized the characteristics of uveitis in 21 children with transiently associated pediatric inflammatory multisystem syndrome (PIMS-TS) related to SARS-CoV-2 [22]. The median age was 11.5 years. Most patients had bilateral anterior uveitis without iris adhesion or high intraocular pressure, and the inflammation lasted for 5–7 days, with a good response to corticosteroids. This prompts clinical pediatricians and ophthalmologists to early identify and treat uveitis, which may cause permanent vision damage due to possible ocular complications.

We found that receiving local or systemic steroid treatment can improve the condition of newly diagnosed or recurrent uveitis. Multiple studies have reported that uveitis can be well controlled through local or systemic steroid treatment. A recent case report showed that a 14-year-old male pneumonia patient was diagnosed with COVID-19-associated bilateral anterior uveitis. The patient’s ocular symptoms improved after treatment with local and systemic corticosteroids [23]. Iriqat et al. reported a case of anterior uveitis that occurred after recovery from COVID-19, which was severe but was relieved after local and systemic steroid treatment [5]. In our study, all of our uveitis patients responded well to local and systemic steroid treatment without complications or relapses during the study period. In addition, none of the patients experienced increased IOP during treatment and all recovered without using antiviral drugs, which may indicate a potential immunogenic etiology for non-granulomatous uveitis. Although the use of corticosteroids in COVID-19 patients is controversial among international experts [24, 25], based on our study observation, we support the timely and appropriate use of corticosteroids in COVID-19 patients with concurrent uveitis, taking into account the severity of uveitis, systemic disease, age, and lens status.

Our study has a limitation in establishing a definitive relationship between uveitis and COVID-19 infection, despite all patients presenting with new-onset or recurrent uveitis symptoms within one month of COVID-19 infection. The overall positive rate of SARS-CoV-2 RNA in ocular fluid is rare, with some studies reporting a failure to detect any RNA in conjunctival swabs [26]. Nevertheless, it is prudent to remain cautious and the potential occurrence of uveitis after COVID-19 infection should be considered. The relative lack of detectable viral RNA in ocular fluid also prompts the question of whether the ocular manifestations of COVID-19 truly result from viral infection in ocular tissues or are part of a spectrum of ocular symptoms accompanying various viral diseases [27]. Furthermore, the sample size in our study was still comparatively small to comprehensively delineate the intricate relationship between COVID-19 infection and uveitis.

## Conclusion

In summary, this retrospective observational study found that COVID-19 infection can cause new-onset and recurrent uveitis. Inflammation can be stabilized with appropriate local or systemic steroid treatment. As the current pandemic continues, this retrospective observational study enhances the understanding of the virus among healthcare workers and highlights the need for further research on the relationship between COVID-19 infection and ocular-related diseases.

## Data Availability

The datasets used and/or analyzed during the current study are available from the corresponding author on reasonable request.

## Abbreviations

COVID-19: 2019 novel coronavirus disease
SARS-CoV-2: severe acute respiratory syndrome coronavirus 2
SUN: Standardization of Uveitis Nomenclature
RT-PCR: real-time reverse transcription polymerase chain reaction
IOP: intraocular pressure
FFA: fluorescein angiography
ICGA: indocyanine green angiography
SD-OCT: spectral domain optical coherence tomography
VKH: Vogt-Koyanagi-Harada syndrome
MEWDS: multiple evanescent white dot syndrome
PPV: pars plana vitrectomy
FAF: fundus autofluorescence
EZ: ellipsoid zone
JIA: juvenile idiopathic arthritis
PSS: Posner-Schlossman syndrome
